# Supplementary material for: Assessment of the quality of life of COVID-19 recovered patients at the Ghana Infectious Disease Centre
Source: PLoS One. 2024 Jul 18;19(7):e0306118. doi: 10.1371/journal.pone.0306118 (PMC11257348; doi:10.1371/journal.pone.0306118)
Supplement: Attachment — Submitted filename: Response to Reviewers_1.docx [file pone.0306118.s002.docx]

The Editor

PLOS ONE

Dear Editor,

**Response to Reviewers: Assessment of the Quality of Life of COVID-19 recovered patients at the Ghana Infectious Disease Centre**

We are grateful to the editor and reviewers for their feedback and helpful recommendations. We are glad to have the chance to resubmit an improved version of our paper for your further evaluation. In the following, we address the reviewers’ remarks and indicate the relevant modifications we applied to the paper. The line numbers we refer to are from the revised manuscript with the track changes off.

**Reviewer #1:**

1. The introduction lacks dept and organization. Researchers should include relevant related literature on similar study done in other parts of the world and also introduce subheadings to improve readability.

**Response:** The introduction has been revised to reflect the depth and inclusion of relevant studies. We however used paragraphs to introduce issues instead of the sub-headings.

2. The writing style will benefit from improvement in areas of conciseness and flow of ideas.

**Response:** This has carefully been looked at and improved.

3. The researchers did not state any inclusion or exclusion criteria for the sample selected. Even though patients were all admitted for COVID-19 facility, their length of stay and COVID-19 related complications were not the same.

**Response:** The inclusion criteria and exclusion criteria employed has been indicated in lines 143-151 of the revised manuscript (without track changes).

4. The researchers did not explain if any power analysis was done to arrive at the sample size.

**Response:** We have provided an explanation on the sample size determination in lines 153-157.

5. Also, the sample selected is skewed towards formally employed (71%) and individuals with tertiary level of education (83%) and thus not representative of the population.

**Response:** Most of the cases that showed up the GIDC which is a (tertiary level facility) were referrals from other facilities typically used by populations with higher socio-economic status and they are more likely to have higher levels of education.

6. The researchers mentioned recall bias but did not state explicitly the data collection process and timelines.

**Response:** The data collection process, particularly for the administration of the EuroQoL tools was to recovered patients who have been attending the post-recovery clinic from one month and above. This has been indicated in the inclusion criteria.

8. Finally, they failed to mention limitations associated with the research design used and its impact on research results and conclusions.

**Response:** The study’s limitations have been included in lines 453-463 of the revised manuscript.

**Reviewer #2:**

1. Title is reflective of study- objectives and rationale.
2. Sample size of 150 adequate for a clinical sample.
3. No reported Cronbach’s alpha for the (EQ-VAS) visual analogue scale.

**Response:** The EQ-VAS is a single measure of quality of life from a self-reported scale and so the use of the Cronbach’s alpha was not applicable unlike the EQ-5D-5L which was derived from multi-dimensional Likert scale measures.

4. No in text references for Discussion Lines 6,7 and 8 - global response by local governments and international bodies such as WHO, UNICEF, and IMF to provide support, financial relief, and public health assistance to curb the devastating nature of the disease.

**Response:** This has been resolved. (Ref to line numbers 326 – 328)

5. Discussion Lines 29-30 Women may also be more worried when it comes to disease with a decreased ability of coping- This needs evidence to back it.

**Response:** This statement has been deleted from the manuscript.

6. Reference list: Rearrange 2, 11, 12, 15, 18, 20, 21, 22, 32, 33, 35. 36

**Response:** The list of references has been revised.

**Reviewer #3:**

Abstract

1. “Differences were not significantly observed for factors such as marital status, residence, ethnicity, and access to rehabilitative centres” does not read well. I suggest “Significant differences were not observed in quality of life for factors such as marital status, residence, ethnicity, and access to rehabilitative centres”.

**Response:** The statement being referred to has been taken out of the abstract

Introduction

2. The introduction is fine with some edits. For example, the sentence in lines 79-82 is unclear in terms of comparison with NCDs and genetic diseases.

**Response:** The Introduction has been extensively revised.

3. The background could touch on some of the potential predictors rooted in relevant literature and possibly a theory relevant to QOL and its predictors.

**Response:** The Background of the Introduction has been revised with additional literature to that effect.

4. The specific objectives addressed in this study need to be stated.

**Response:** This has been included in the introduction (Ref to line numbers 125 – 127)

Materials and Methods

5. Lines 154-156. “Linear regression models were used to assess the mean differences” Linear regression models are used to test relationships/predictions. Check and clarify.

**Response:** This statement has been deleted from the manuscript. The regression methods used have also changed and explained accordingly.

Results and Discussion

6. Authors need to justify the analysis in Table 5 as it is a repetition of analysis presented in Table 4.

**Response:** The analysis in Table 4 shows the mean QoL scores and compares them by subdomains of EQ-5D-5L as well as overall with Kruskal-Wallis tests for each independent factor. Table 5 in its current form is from adjusted quantile regression using the overall median QoL scores and the reason for this has been explained in line numbers 265-266.

7. The discussion needs to be rewritten to focus on the key findings that addressed the objectives of the study.

**Response:** The discussions have been revised accordingly.

8. No contextual reasons were adduced for the high QOL scores among the study participants beyond global and local efforts (which were not duly discussed). What about possible roles of family and significant others in the Ghanaian context of care?

**Response:** We believe the revised discussions will resolve this.

9. “This study found four main predictors of quality of life in the Ghanaian patient post-recovery from COVID-19. These were sex, persistent symptoms, worsening comorbidities, and development of complications post-COVID-19 infection”. This is not accurate as there are differences in QOL across several factors.

**Response:** There were multiple factors independently associated with QoL from Table 4, but the analysis shown in Table 5 which takes care of confounding effects using the medians provides a more robust determination of these significant factors.

10. The discussion needs to be focused and structured according to the study objectives.

**Response:** The discussions have been revised accordingly.

Thank you
