# Supplementary material for: Assessment of the quality of life of COVID-19 recovered patients at the Ghana Infectious Disease Centre
Source: PLoS One. 2024 Jul 18;19(7):e0306118. doi: 10.1371/journal.pone.0306118 (PMC11257348; doi:10.1371/journal.pone.0306118)
Supplement: Attachment — Submitted filename: Response to Reviewers_2.docx [file pone.0306118.s003.docx]

The Editor

PLOS ONE

Dear Editor,

**Response to Reviewers: Assessment of the Quality of Life of COVID-19 recovered patients at the Ghana Infectious Disease Centre**

We are grateful to the editor and reviewers for their feedback and helpful recommendations. We are glad to have the chance to resubmit an improved version of our paper for your further evaluation. In the following, we address the reviewers’ remarks and indicate the relevant modifications we applied to the paper.

**Additional editor comments**

In addition to the other comments, the authors should further address this comment by adding a sentence in the limitation section. "5. Also, the sample selected is skewed towards formally employed (71%) and individuals with tertiary level of education (83%) and thus not representative of the population.

Response: Most of the cases that showed up the GIDC which is a (tertiary level facility) were referrals from other facilities typically used by populations with higher socio-economic status and they are more likely to have higher levels of education."

**Response:** We thank the editor for the suggestion. The limitations section has been updated accordingly. Lines 448-452

**Reviewer 3**

Reviewer #3: Lines 32-33: The sentence does not read well.
Line 42: the beta value should be checked (9.40 [-14.19, -4.61]) because the 95%CIs are all in the negative.

**Response:** We thank the reviewer for noticing these errors. Lines 32-33 and 42 have been revised. The beta value is -9.40.

**Authors additional revisions**

We also included additional information on the sampling section to improve clarity. Lines 148-152

Thank you
